# Detection of High-Risk Human Papillomavirus (HPV), p16 and EGFR in Lung Cancer: Insights from the Mediterranean Region of Turkey

**DOI:** 10.3390/v16081201

**Published:** 2024-07-26

**Authors:** Arsenal Sezgin Alikanoğlu, İrem Atalay Karaçay

**Affiliations:** 1 Pathology Department, Antalya Education and Research Hospital, Health Sciences University, Antalya 07100, Turkey; 2 Pathology Department, Alanya Alaaddin Keykubat University, Antalya 07400, Turkey

**Keywords:** lung cancer, human papillomavirus (HPV), polymerase chain reaction (PCR), viral oncology, p16 protein

## Abstract

Human papillomavirus (HPV) is an oncogenic DNA virus that plays a role in different cancer types. The aim of this study was to detect the prevalence and types of HPV and its relation with p16, EGFR and clinical findings in lung cancer. HPV and EGFR detection and genotyping of HPV were performed by polymerase chain reaction (PCR) and p16 by immunohistochemistry. Fifty lung cancer patients and seven patients with non-neoplastic lung disease were enrolled in this study. HPV was positive in 78% (39/50) of lung cancer cases. HPV 51 was the most frequent type, followed by HPV 16. Moreover, p16 was positive in 24% (12/50) of the cancer patients, and all of these patients were HPV-positive, while 27 HPV-positive patients showed no p16 expression. There was no relationship between HPV infection and p16 (*p* = 0.05), gender (*p* = 0.42), age (*p* = 0.38), or smoking history (*p* = 0.68). Although not statistically significant, the HPV prevalence was found to be higher in cancer patients compared to non-neoplastic patients. The prevalence of HPV in lung cancer varies across different studies, which may be due to differences in the detection methods, number of patients, geographic regions, and vaccination status. Further studies are necessary to understand the role of HPV in lung cancer pathogenesis.

## 1. Introduction

Lung cancer is the leading cause of cancer death in both men and women worldwide. Smoking ranks first among the etiological factors that cause the development of lung cancer [1,2,3]. The cases of lung cancer detected in non-smokers are more often seen in females and in Asian countries. Furthermore, these cases have different molecular characteristics compared to the cases of lung cancer in smokers. Genetic susceptibility, radiation, environmental pollution, occupational exposure, and infectious agents, especially those of viral origin, can be counted among the other factors that play a role in lung carcinogenesis, apart from smoking [4,5,6].

It is known that about 10–15% of cancers seen in humans all over the world are caused by Epstein–Barr virus, hepatitis B or C virus, human T-lymphotropic virus-1, human papillomavirus (HPV) and Merkel cell polyomavirus. The viruses can promote cancer as carcinogens or promoters.

HPV is a non-enveloped, small, double-stranded circular DNA virus and has “low-risk” or “high-risk” types, which are defined according to their relation with cancer development. HPV is known to dysregulate the cell cycle at the transition from the G1 to S phase and to promote DNA synthesis for viral replication. The expression of the most important viral oncoproteins, E6 and E7, is considered a first step in carcinogenesis since these oncoproteins inactivate the two important products of tumor suppressor genes p53 and retinoblastoma protein (pRb), respectively [7]. P16 is also a tumor suppressor protein that functions as an inhibitor of cyclin-dependent kinase 4 (CDK4) and regulates the cell cycle. Loss of p16, as reported in a variety of tumors, including lung cancer, causes phosphorylation of Rb, which ends up with uncontrolled cell proliferation [8,9,10].

The link between HPV and bronchial lesions was first established in the 1970s by Rubel and Reynold, who found that there is cytological and histological similarity between condyloma acuminatum and squamous cell papilloma of the respiratory tract. They detected koilocytes, which are characteristic of HPV infections in the sputum samples of patients with benign bronchial lesions [11]. There are also other studies presenting condylomatous histological changes in bronchial epithelium and bronchial squamous cell carcinoma, similar to the changes seen in the genital tract [4,12,13].

After these detections, the relationship between HPV infection and lung cancer has been investigated in several studies until today. These studies revealed a great difference in the HPV infection rate in lung cancer (0–78.3%) in different regions of the world. It is suggested that this wide range may be due to the difference in sensitivity and specificity of the methods used for HPV genotyping, the number of types of HPV analyzed, the diagnostic criteria, the number and characteristics of patients and the ethnicity of patients [1,3,14,15,16,17,18,19].

Epidermal growth factor receptor (EGFR) is a receptor tyrosine kinase that affects some signaling pathways in cell proliferation. Mutations in EGFR may cause uncontrolled growth and proliferation of cells, especially in non-small-cell lung cancer (NSCLC). EGFR mutations are detected more frequently in women, non-smokers, adenocarcinoma, and Asian populations [20,21,22,23]. The most commonly detected mutations in EGFR in NSCLC are exon 19 deletion (small in-frame deletions in exon 19) and L858R point mutation (amino acid substitution (leucine to arginine) at codon 858 in exon 21) [20,21]. EGFR tyrosine-kinase inhibitors (gefitinib, erlotinib) are considered the standard first-line treatment for patients with EGFR mutations and therefore it is recommended that screening for EGFR mutations should be a part of the routine clinical practice for NSCLC patients [20,23]. Previous studies demonstrated that HPV infection was found more frequently in lung adenocarcinoma patients with EGFR gene mutations than in patients without mutations, suggesting that the viral protein E6 regulates the inhibitors of apoptosis of the EGFR/PI3K/AKT signaling pathway [22,23].

In this study, we aimed to investigate the prevalence of HPV and its genotypes and the relation with EGFR mutations, p16 protein expression and clinicopathological findings in lung cancer.

## 2. Materials and Methods

### 2.1. Patient Selection and Data Collection 

This study included 50 patients who had a histopathologic diagnosis of primary lung carcinoma in lobectomy specimens and 7 patients who had surgery for a non-tumoral lung pathology (bullous disease, infection) at a tertiary-level hospital between 1 January 2013 and 1 January 2019. None of the patients received HPV vaccination. This study was performed in accordance with the ethical standards of the Declaration of Helsinki, 2013. The study was approved by the Ethics Committee of Health Sciences University, Antalya Education and Research Hospital (date and register number: 2019-242, 19/9). Hematoxylin and eosin sections of the cases were obtained from the archive and examined histologically by the authors (A.S.A. and İ.A.K.). The demographic and clinical characteristics of the patients, including age, gender, smoking history (the patients who self-reported as a current or former smoker and had smoked ≥100 cigarettes in their lifetime were accepted as “smoker”, based on the criteria proposed by the Centers for Disease Control and Prevention), and pathological data were obtained through patient records and pathology reports from the hospital database. The non-metastatic patients (42/50) received adjuvant chemotherapy, while the patients without EGFR and anaplastic lymphoma kinase (ALK) mutation received palliative platinum-based systemic chemotherapy at a metastatic setting. Eight of the patients had distant metastasis. According to the frequency of occurrence, metastases were detected in the brain, costa and liver.

### 2.2. DNA Extraction 

DNA was extracted from the formalin-fixed paraffin-embedded blocks of 57 patients for HPV analysis and 50 patients for EGFR analysis. Eight tissue sections of 10 µm thickness were used per patient for DNA extraction. DNA was extracted and deparaffinized from the sections using the QIAamp DNA FFPE Tissue kit (Qiagen, Hilden, Germany) according to the manufacturer’s instruction for real-time PCR analysis of HPV and EGFR. In order to avoid contamination, precautions were taken, including extensive cleaning of the work area, changing the microtome blades and cleaning of the microtome after cutting each sample, performing extraction of DNA in tumoral and non-tumoral samples separately and using appropriate protection equipment.

### 2.3. HPV DNA Detection and Genotyping

Detection and genotyping of human papillomavirus (16, 18, 31, 33, 35, 39, 45, 51, 52, 56, 58, 59, 66, 68) were performed using the HPV Genotypes 14 Real-TM Quant kit (Nuclear Laser Medicine, Milan, Italy), which was based on two major processes: isolation of DNA from specimens and multiplex real-time amplification of 4 PCR tubes for each sample, each tube amplifying “16-18-31-IC”, “39-45-59-IC”, “33-35-56-68” and “51-52-58-66”. For each sample, negative and positive clinical samples were used as controls. The HPV Genotypes 14 Real-TM Quant kit contains the internal control (human beta-globin gene), which allows control of the presence of cellular material in the sample in order to avoid false-negative results.

HPV DNA amplification was carried out in the real-time PCR cycler (Rotor-Gene™ 3000/6000/Q (Qiagen, Hilden, Germany), and for the quantitative analysis, Microsoft^®^ Excel HPV Genotype 14 Real-TM.xls, ver. 09.09.21 (Sacace Biotechnologies^®^, Como, Italy) was used according to the enclosed instructions. 

### 2.4. EGFR Mutation Detection

The extracted DNA samples were assessed using real-time PCR (Rotor-Gene™ 3000/6000/Q, Qiagen, Hilden, Germany) with the Easy EGFR Real Time PCR kit (Diatech Pharmacogenetics, Jesi, Italy) following the manufacturer’s protocol. Each DNA sample was analyzed for mutations on exon 18 (G719X), exon 19 (ex19del), exon 20 (T790M, S768I, ex20ins), and exon 21 (L858R, L861Q). A positive control was included with the kit, and distilled water was used as the negative control.

### 2.5. p16 Immunohistochemical Staining

For the detection of p16, monoclonal mouse anti-human antibody (clone INK4A, Clone IHC116, 1:200 dilution, GeneAb, Richmond, BC, Canada) was used according to the manufacturer’s suggested protocol, using the automated staining system Bench-mark XT (Roche/Ventana Medical Systems, Tucson, AZ, USA). Deparaffinization at 75 °C was followed by antigen retrieval by CC1 buffer for 64 min. Incubation lasted for 44 min at 36 °C. Diaminobenzidine was used as a chromogen. After washing, the slides were placed in differently graded alcohol solution and xylene and finally mounted with entellan. Cervical cancer and parathyroid tissue were used as positive and negative controls, respectively. The slides were evaluated by the authors (A.S.A., İ.A.K.). Nuclear and cytoplasmic staining in ≥10% of the tumor cells was accepted as positive, while <10% was considered as negative for p16 [8].

### 2.6. Statistical Analysis

Statistical analyses were carried out using IBM SPSS Statistics for Windows, version 23.0 (IBM Corp., Armonk, NY, USA). The descriptive findings were presented as the mean ± standard deviation (SD) for the continuous data, and as the frequency and percentage for the categorical data. The normality assumptions were controlled by the Shapiro–Wilk test. Categorical data were analyzed by the Pearson chi-square test and Fisher’s exact test. Student’s *t*-test was used for analysis of the normally distributed numerical data. Two-sided *p* values < 0.05 were considered statistically significant.

## 3. Results

### 3.1. Clinicopathological Characteristics of Patients 

This study included 57 patients with a mean age of 63.2 ± 2.8 (range 27–83 years). In this study, the male gender prevailed (84.2%) and almost 60% of patients had a history of smoking. Thirty of the male patients and four of the female patients were smokers. All the patients with non-neoplastic disease were non-smokers. The two most common histological tumor types were squamous cell carcinoma (54.4%) and adenocarcinoma (22.8%).

The clinicopathologic characteristics of the patients are summarized in Table 1.

### 3.2. HPV Infection, Prevalence of HPV Types, and Their Relationship with Clinicopathologic Parameters

In total, 75.4% (43/57) of the patients were HPV-positive. The HPV positivity rate was 57.1% (4/7) in the non-neoplastic samples and 78% (39/50) in the neoplastic samples. All the adenocarcinomas (13/13, 100%) and 64.5% (20/31) of the squamous cell carcinomas showed positivity for high-risk HPV DNA. HPV 51 was the most frequent HPV type (49.1%, n = 27), followed by HPV 16 (43.9%), HPV 31 (15.8%), and HPV 18 (12.3%). Among the smokers, the HPV positivity rate was found to be 73.5% (25/34), and 28% (14/50) of the cancer patients were non-smokers and HPV-positive. In terms of the histopathological diagnosis and HPV status, the frequency of adenocarcinoma was higher in the HPV-positive cases.

**Figure 1:**
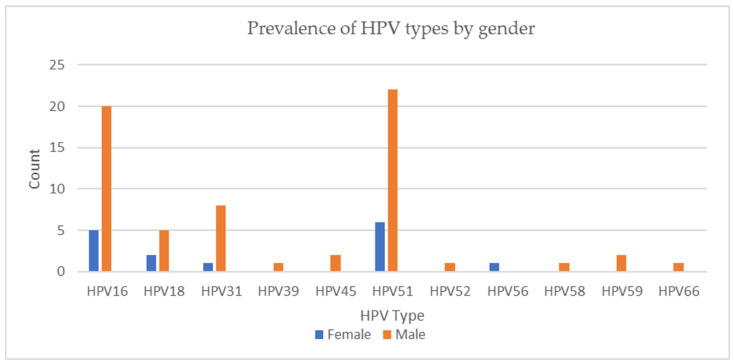
Prevalence of HPV types by gender.

**Figure 2:**
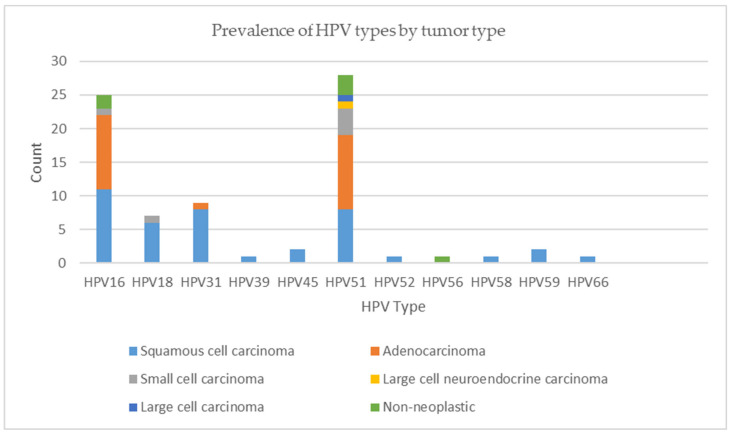
Prevalence of HPV types by tumor type.

**Figure 3:**
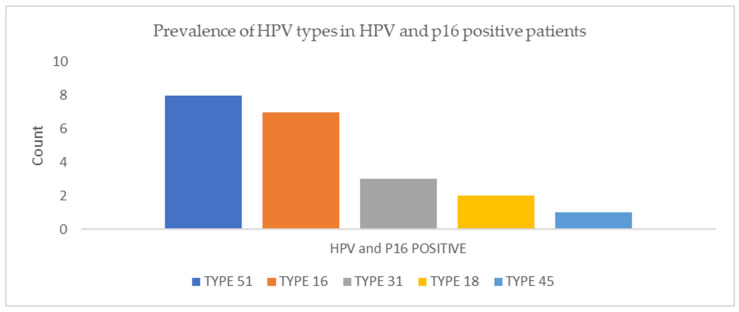
Prevalence of HPV types in HPV- and p16-positive patients.

The prevalence of the HPV types by gender and tumor type are represented in Figure 1 and Figure 2. Higher rates of HPV 51 and 56 positivity were detected in women; however, there was no statistically significant relationship between HPV infection and gender (*p* = 0.42), age (*p* = 0.38), or smoking history (*p* = 0.68). HPV 16 positivity was more common in adenocarcinomas (*p* = 0.006). Furthermore, HPV 51 was more frequent in adenocarcinomas and other histological types of carcinomas, including large- and small-cell carcinoma and large-cell neuroendocrine carcinoma (*p* < 0.001), and it was the only type detected in the histological types of carcinomas other than adenocarcinoma and squamous cell carcinoma. When the HPV and p16 positive tumors were analyzed as one category, HPV 51 was the most frequent type, followed by HPV 16, as shown in Figure 3.

**Figure 4:**
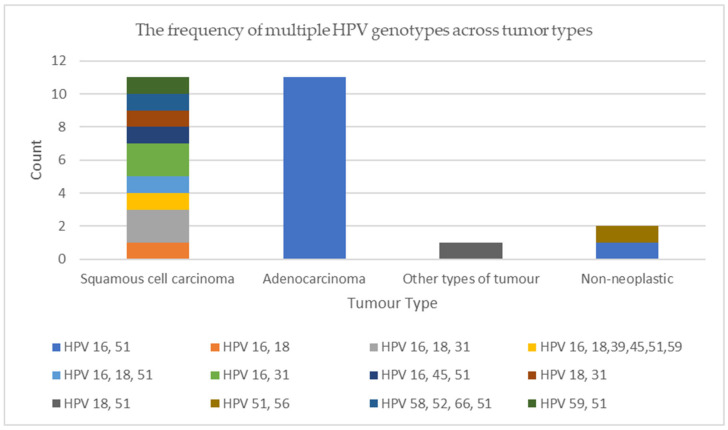
The frequency of multiple HPV genotypes across tumor types.

Most samples (58.1%, 25/43) contained multiple HPV types, with HPV16 and HPV51 being the most prevalent. Figure 4 displays the occurrence of multiple HPV genotypes among the different types of tumors and non-neoplastic lung tissue.

### 3.3. Presence of EGFR Mutation and Its Relation with HPV 

EGFR mutation was detected in 3/50 (6%) of lung cancer cases, and all the cases with EGFR mutation revealed exon 19 deletion. All the EGFR-mutated patients had a histopathological diagnosis of adenocarcinoma and revealed multiple infection of HPV types 16 and 51, and two of them were smokers. There was no significant relationship between HPV positivity and the presence of EGFR mutation in the lung cancer cases (*p* > 0.999). 

### 3.4. Detection of p16 by Immunohistochemistry

The expression of the p16 protein was detected in 12 of the 50 (24%) cancer patients. HPV was positive in 100% of the p16-positive cases. A total of 11 cases were both p16-negative and HPV-negative. The number of HPV-positive and p16-negative cases was found to be 27. There was no significant relationship between HPV status and p16 protein expression (*p* = 0.05), as shown in Table 2. The p16 expression showed variation in the different histological types of tumors. The highest expression was found in squamous cell carcinoma (7/12), followed by small-cell carcinoma (3/12), large-cell adenocarcinoma (1/12) and adenocarcinoma (1/12), as shown in Table 1.

**Figure 5:**
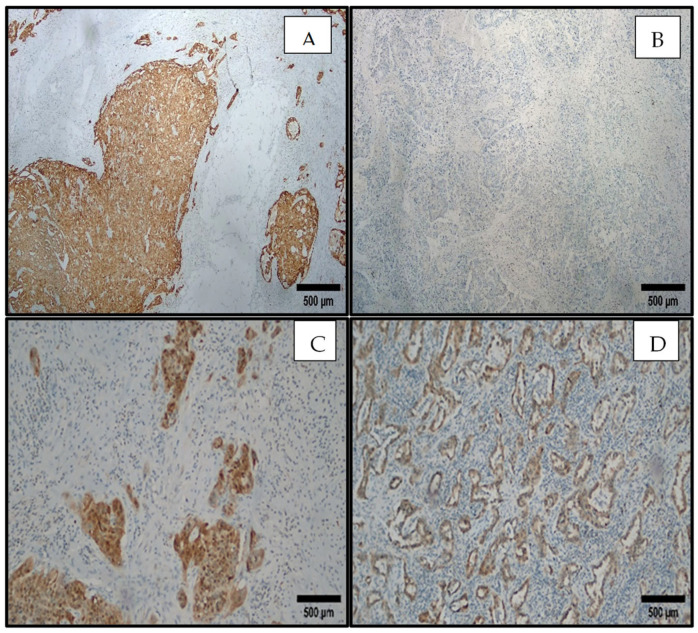
p16 staining, immunohistochemistry, Nikon Ci-L light microscope. (Nikon Corporation, Yokohama, Japan) (**A**) Positive in cervix cancer as a positive control, ×40. (**B**) Negative in lung adenocarcinoma, ×40. (**C**) Positive in lung squamous cell carcinoma, ×100. (**D**) Positive in lung adenocarcinoma, ×100.

Figure 5 shows the immunohistochemical expression of p16 in the positive control and cancer tissue samples. 

## 4. Discussion

Since lung cancer is the leading cause of cancer death worldwide, studies on the etiological factors and carcinogenesis are important, especially for guiding prophylactic therapy with vaccines and targeted therapy. Although there is some supporting evidence concerning the relation between HPV infection and lung carcinogenesis, the subject is still controversial, and because of this, studies on this issue are ongoing. 

The rate of HPV positivity in lung cancer reported in several studies is between 0 and 78.3%. Some of the studies report that there is a close relationship between HPV and lung cancer [3,14,24,25,26], while some of them report the very low prevalence of HPV in lung cancer [1,2,27,28,29]. HPV seems to be more associated with lung cancer in certain geographical regions of the world [6]. In many studies, it is evidenced that the prevalence of HPV in lung cancer is higher in Asian countries than on the other continents [15,30,31,32,33,34,35]. Furthermore, the HPV prevalence rate ranging from 0% to 61% in different studies conducted in Greece indicates that the results can be discrepant even within the same country [14,27].

In previous studies, the HPV positivity rate was found to be higher in cancer patients than in non-neoplastic controls [15,26,36]. The most recent meta-analysis study reported the prevalence difference of HPV to be 22% in lung cancer compared to control cases [24]. In our study, HPV positivity was detected in 78% of the cancer group and 57% in the non-neoplastic group. The prevalence difference was found to be 21%, similar to the meta-analysis study mentioned above.

To date, few studies have investigated the prevalence of HPV in lung cancer in Turkey. The rate of HPV positivity reported in these studies is between 1.54 and 11.5% and the most commonly detected HPV type is HPV 16/18 [37,38,39]. The differences in the sample types and sizes, as well as the HPV detection/genotyping methods used, may be the reason for the different results.

The high rate of HPV prevalence detected in our study in both cancer and non-neoplastic tissue may be due to factors including the vaccination status and the number of the patients selected from a single region. The FDA-approved HPV vaccine has been on the Turkish market since April 2007. But, in Turkey, the HPV vaccine is not included in the national vaccination program, and people who want to be vaccinated are vaccinated at their own expense. None of the patients included in our study were vaccinated. 

In previous studies, there are conflicting results regarding the association between smoking and HPV infection. Some studies have shown that the interaction of smoking and HPV induces viral oncoproteins [40] and that the majority of HPV-positive lung cancers are smokers [36], whereas others concluded that smoking is an independent factor in lung cancer [41]. It is suggested that some other factors, such as the number of lifetime sexual partners, other lifestyle habits and additional molecular alterations may play a role in HPV-related carcinogenesis in smokers [42]. In our study, the rate of HPV positivity was higher in females and close between smokers and non-smokers with a little difference (73.5% and 78.2%), and no statistically significant difference was found between HPV infection, age, gender, and smoking history, similar to the results of some other studies [1,27].

The relation between the presence of HPV and the histopathological type of lung cancer is discrepant between studies. Some studies have observed the higher prevalence of HPV in squamous cell carcinoma [3,10,14,15,24], while others have found a greater presence in adenocarcinomas [9,32,43,44]. Our findings support the latter. The most frequently detected HPV types worldwide are types 16 and 18 [3,24,26,29], while the other commonly detected high-risk types are known to be types 31 and 33 [31]. In our study, the two most commonly detected types were type 51 (49.1%) and type 16 (43.9%), followed by type 31 (15.8%) and type 18 (12.3%). 

Although some studies found that HPV 16/18 is significantly associated with squamous cell carcinoma, some reported that there is no statistically significant relation between HPV 16/18 and the histopathological subtypes of cancer [3,26]. In a study by Baba et al., HPV type 16 was the most frequently detected type in both adenocarcinoma and squamous cell carcinoma [43]. In our study, HPV 16 positivity was more common in adenocarcinomas (*p* = 0.006). These different results may be due to the prevalence of the histopathological type of cancer in the region, ethnicity, sample size of the study and other variables [3]. 

Furthermore, some studies revealed that HPV infections were more commonly detected in patients with EGFR mutations [22,23,24,25,45,46]. It is assumed that the EGFR/PI3K/AKT pathway may play an important role in HPV-associated lung carcinogenesis in EGFR-mutated patients [22,23,47]. In our study, EGFR mutation was detected in 6% (3/50) of the patients with cancer and all of the EGFR-mutated patients had a histopathological diagnosis of adenocarcinoma and multiple HPV infection with HPV types 16 and 51. There was no significant relationship between HPV positivity and the presence of EGFR mutations in the lung cancer cases (*p* > 0.999). This result may be due to the small number of EGFR-mutated patients detected in this study. 

Moreover, p16, which is known as one of the regulators of the cell cycle, is found to be inhibited in several cancer types through methylation and deletion. In dysplastic and neoplastic cervical lesions, and oropharyngeal squamous cell carcinomas, the transcription of the p16 gene is inhibited by HPV, resulting in abnormal expression of the p16 protein, dysregulation of the cell cycle and tumorigenesis [48]. However, it is suggested that the well-established relationship between HPV and p16 in cervical and oropharyngeal cancer may not be the same in lung cancer. Not all HPV-infected patients who are expressing its oncoproteins exhibit significant changes in p16 expression [9,49,50]. One of the aims of our study was to search the relationship between HPV and p16 in lung cancer. We found that p16 expression was positive in 24% (12/50) of lung cancer patients and all (12/12) of the p16-positive patients were HPV-positive while none of the HPV-negative patients showed p16 expression and 30.8% (12/39) of the HPV-positive patients were p16-positive. In our study, there was no significant relationship between HPV status and p16 protein expression (*p* = 0.05) in consistent with the results some other studies in the literature [9,49,50]. 

There were also discrepant results concerning p16 expression in different types of lung tumors in the literature, with some studies reporting higher expression in adenocarcinomas while others found it to be higher in SCCs [9,10]. In our study, we found p16 expression to be higher in squamous cell carcinomas than adenocarcinomas. The number of patients, the distribution of the tumor types and the p16 detection method may be the reasons behind the discrepancy in the results.

Although there may be some possible limitations in our study, such as the small number of patients and lack of follow-up data for survival analysis, it contributes to the literature by providing information about the prevalence of HPV, the distribution of HPV types, and its relationship with p16 in lung cancer patients from the Mediterranean region of Turkey.

## 5. Conclusions

In conclusion, our study demonstrated the presence of HPV in lung cancer (39/50, 78%), but we think that more evidence is needed to prove the relationship between the presence of HPV and lung carcinogenesis. HPV infection may be one of the factors playing a role in the development of lung cancer. However, further studies using reliable methods to search for HPV in a larger number of lung cancer patients across different geographic regions are necessary to understand the role of HPV in lung cancer pathogenesis.

## Figures and Tables

**Table 1:** The clinicopathological characteristics of the patients and their HPV status.

Characteristic		HPV Positive (n)	HPV Negative (n)	HPV and p16 Positive (n)
Age (years)	≤62	20	1	3
	>62	23	13	9
Gender	Female	8	7	3
	Male	35	7	9
Smoking history	Smoker	25	9	6
	Non-smoker	18	5	6
Histopathological diagnosis	Squamous cell carcinoma	20	11	7
	Adenocarcinoma	13	0	1
	Other tumors	6	0	4
	Non-neoplastic	4	3	- *
EGFR status	Wild type	36	11	11
	Mutated	3	0	1
	Not applied	4	3	0

n: number of patients, * p16 immunohistochemistry was not applied in non-neoplastic patients.

**Table 2:** HPV rates in the p16-positive and -negative groups.

		HPV-Positive (n = 39)	HPV-Negative (n = 11)	*p* *
P16	Positive	12 (30.8)	0 (0)	0.05
	Negative	27 (69.2)	11 (100)	

* Fisher’s exact test, n (%).

## Data Availability

Data are contained within the article.
